# Asymmetric charge balanced waveforms direct retinal ganglion cell axon growth

**DOI:** 10.1038/s41598-023-40097-6

**Published:** 2023-08-14

**Authors:** M. G. Peng, E. Iseri, A. Simonyan, P. Lam, T. Kim, S. Medvidovic, J. Paknahad, M. Machnoor, G. Lazzi, K. K. Gokoffski

**Affiliations:** 1 Department of Ophthalmology, Keck School of Medicine, USC Roski Eye Institute, University of Southern California, Los Angeles, CA USA; 2 Department of Electrical and Computer Engineering, Viterbi School of Engineering, University of Southern California, Los Angeles, CA USA

**Keywords:** Adult neurogenesis, Cellular neuroscience

## Abstract

Failure to direct axon regeneration to appropriate targets is a major barrier to restoring function after nerve injury. Development of strategies that can direct targeted regeneration of neurons such as retinal ganglion cells (RGCs) are needed to delay or reverse blindness in diseases like glaucoma. Here, we demonstrate that a new class of asymmetric, charge balanced (ACB) waveforms are effective at directing RGC axon growth, in vitro, without compromising cell viability. Unlike previously proposed direct current (DC) stimulation approaches, charge neutrality of ACB waveforms ensures the safety of stimulation while asymmetry ensures its efficacy. Furthermore, we demonstrate the relative influence of pulse amplitude and pulse width on the overall effectiveness of stimulation. This work can serve as a practical guideline for the potential deployment of electrical stimulation as a treatment strategy for nerve injury.

## Introduction

Since the 1920s, scientists have struggled to develop electric field (EF) application into a technology to enhance endogenous repair mechanisms in the central nervous system (CNS) to direct axon regeneration^1,2^. The premise for this approach stems from work showing that EFs are naturally generated by the body and play a pivotal role in directing tissue patterning during development^3^ and wound healing after injury^4^. In the case of the optic nerve, immediate application of EFs after nerve transection promoted 1.5-fold more survival of retinal ganglion cells (RGCs), the cells whose axons make up the optic nerve, over untreated controls^5^. Electrical activity has also been shown to enhance RGC axon growth in response to brain derived nerve factor (BDNF) in vitro^6^. Our group has shown that EFs not only promote axon growth, but can also control the direction in which RGC axons grow^7^. In the presence of an EF with an amplitude between 1 and 2 V/cm, RGC axons project towards the negative electrode. In fact, the direction of RGC axon growth can be readily manipulated by changing the location of the negative electrode, suggesting that EFs may be exploited to direct target-specific axon growth—a quality of significant importance when designing therapies for highly structured tissues such as the central nervous system (CNS).

Although these compelling studies suggest that EFs could be developed into a technology to help direct regeneration of damaged axons, electrical stimulation has largely failed to translate clinically due to dependence on direct current (DC). DC stimulation leads to charge accumulation, resulting in tissue damage with high voltage or prolonged exposure^8^. Alternating current (AC) is a natural and safer alternative because it pairs a positive pulse with a symmetric negative pulse—thus injecting a net charge of zero. AC application, however, has been associated with only mild visual gains in clinical application^9^ and does not promote directional growth^10^.

A potential solution lies in hybrid currents or asymmetric charge-balanced (ACB) waveforms which, like AC, pair anodic (positive) pulses with cathodic (negative) pulses but differ from AC in that the amplitudes and pulse durations of the anodic and cathodic pulses are asymmetric. We hypothesize that this asymmetry will be effective at driving axon growth. Designing ACB waveforms, however, is currently limited by our rudimentary understanding of the relative contribution of different waveform parameters including pulse amplitude, pulse width, and stimulation frequency on controlling cellular behavior. Here, we performed a systematic investigation into the optimal level of asymmetry in pulse duration and amplitude per phase needed to effectively promote directional RGC axon growth in vitro. Our investigations revealed that pulse duration has a more profound influence on RGC axon growth than pulse amplitude. Using this information, we tested various types of ACB waveforms and found that ACBs with higher asymmetry were more effective at directing axon growth than ACBs with lower asymmetry. Symmetric pulses were *not* effective at directing galvanotaxis.

## Methods

### Retinal ganglion cell purification

The use of animals was in accordance with the ARRIVE guideline and the Association for Research in Vision and Ophthalmology (ARVO) Statement on the use of animals for research and was approved by the Ethical Committees at the University of Southern California^11^. CD1 mice were obtained from Charles River Laboratories. Post-natal day 0–1 (P0-P1) CD1 pups were euthanized according to institutional board protocol. Globes were enucleated and placed in ice cold DMEM:F12 media (Gibco, Langley, OK, 11320-082). Retinas were isolated and RGCs were purified using a magnetic-bead separation method that has been previously described^12^. Briefly, retinas were digested for 4–5 min at 37 °C in Hanks Balanced Salt Solution (HBSS; VWR, Radnor, PA 45000-458) containing 20 U/ml papain and 0.005% DNase I (Worthington Biochemicals, Lakewood, NJ). Digestion was neutralized with ovomucoid and 0.005% DNase I (Worthington Biochemicals); the retina was then triturated. Dissociated cells were coated with rabbit anti-mouse Thy1.2 antibody conjugated to micrometal beads (130-049-101, Miltenyi Biotech, Auburn, CA) for 15 min at room temperature (RT) in MACS buffer (phosphate-buffered saline with 0.5% bovine serum albumin and 2 mM EDTA; Miltenyi Biotech, Auburn, CA). RGCs were then purified from the cell suspension using a single metal column with and without a magnetic field, sequentially. Purified RGCs were then quantified and then diluted to 0.3 × 10^6^ RGCs/ml in media (see below). 1 ml of cells was then plated onto an electrotaxis chamber (see below) then incubated at 37 °C overnight for 12 h. Culture purity was assessed by quantifying the percentage of total cells that stained positive for (+) RBPMS at × 10 magnification (see below). RGC purity was found to be 85% ± 3% (n = 3 separate cultures, 5030 total cells counted), slightly lower than Gao et al. who passaged cells through two columns sequentially^13^.

Tissue culture media was made with 500 ml Neurobasal A medium (Thermoscientific, Waltham, MA, 10888022), 2.5 ml 100X l-glutamine (Thermoscientific, 25030081), 10 ml B27 (Gibco, 17504044), and 5 ml penicillin–streptomycin. 45 ml of this media was combined with 5 ml of 2.75% methylcellulose in 1 × IMDM and 10 mM HEPES. Media was supplemented with 50 ng/ml BNDF (Peprotech, Rocky Hill, NJ 450–02), 50 ng/ml CTNF (Peprotech, 450–13), 5 uM Forskolin (StemCell Technologies, Cambridge, MA, 72114) and 5 ug/ml insulin (Sigma, St. Louis, MO 91077C).

### Electrotaxis chamber preparation

**Figure 1:**
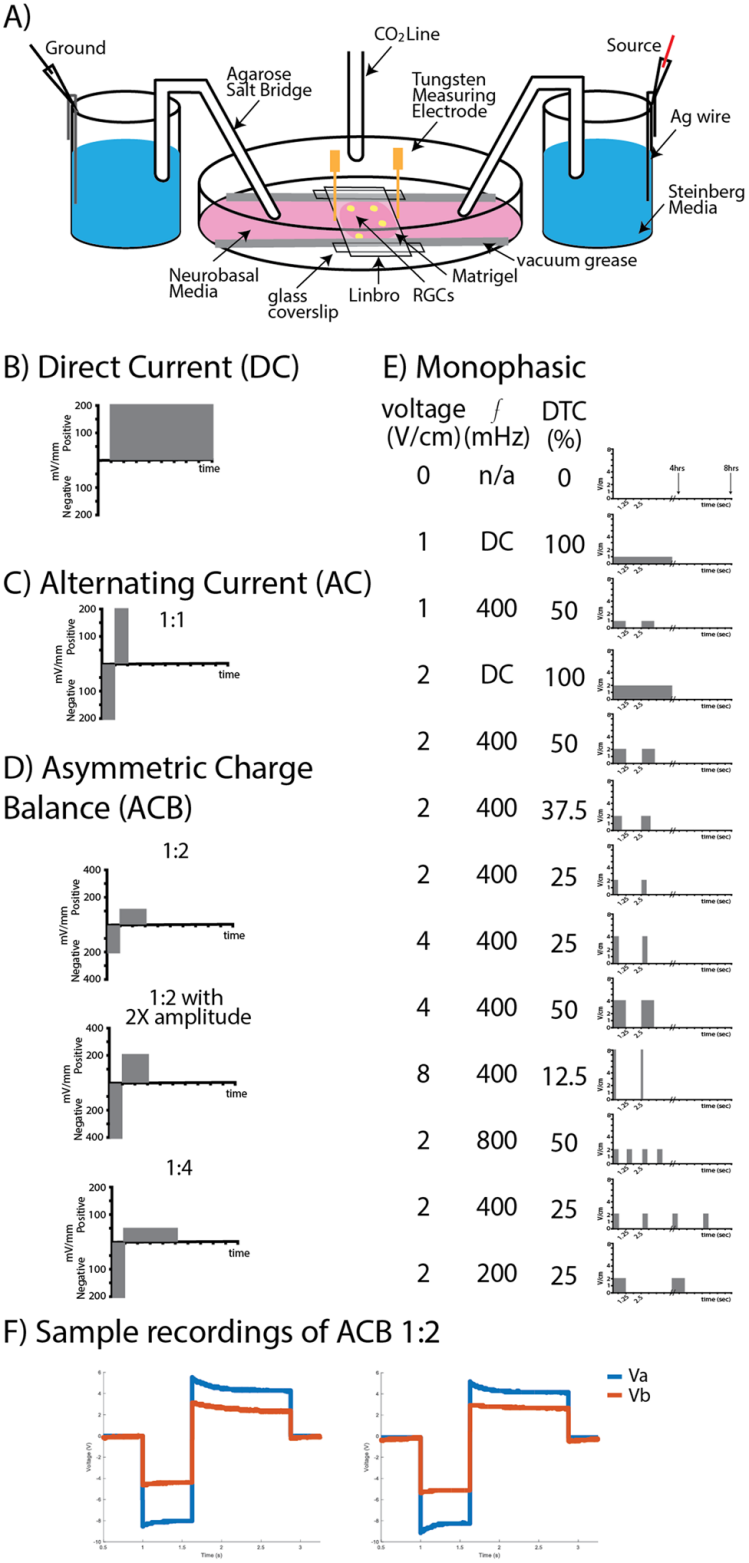
Schematic of Electrotaxis Chamber and Waveforms Tested. (**A**) An electrotaxis chamber was built onto a 100 mm tissue culture plate previously coated with Matrigel. Purified RGCs (yellow) were seeded onto the plate and agarose salt bridges were used to deliver current to the electrotaxis chamber. Representative schematic of (**B**) direct current (DC), (**C**) alternating current (AC), (**D**) asymmetric charge balanced (ACB) waveform, and (**E**) monophasic pulse waveforms studied. DTC, duty cycle. (**F**) An example of voltages recorded across an electrotaxis chamber (Va-Vb) stimulated with ACB waveform 1:2. The left plot was recorded at the start of stimulation while the right plot was recorded after four hours of stimulation.

100 mm tissue culture plates (Genesee San Diego, CA 25-202) were UV sterilized for 20 min. A sterile glass tissue culture insert was placed in the center of the plate (Fig. 1 and Fig. S1). Plates were then coated with Matrigel (VWR, Radnor, PA RBD354277). Matrigel was allowed to settle for 60 min in a 37 °C incubator. Residual, unbound matrigel was then removed and purified RGCs were seeded onto the plates and incubated overnight at 37 °C. The next day, the tissue culture insert was removed and vacuum grease was applied to the plate to attach sterile cut glass coverslips to build the walls of the electrotaxis chamber, centered around the seeded cells. Pre-cut Linbro plate sealer was then placed over the RGCs and attached to the chamber walls, serving as the roof of the chamber. When fully assembled, the dimensions of the chamber through which current was passed measured 30 mm × 10 mm × 0.5 mm.

### Stimulation experiments

EFs were applied as described previously^7^. Briefly, tissue culture plates were placed in a gas/temperature chamber-controlled inverted Axio Observer 7 microscope (Carl Zeiss, Oberkochen, Germany). Agarose salt bridges were used to connect silver electrodes in beakers of Steinberg’s solution to pools of culture media on either side of the electrotaxis chamber to prevent diffusion of electrolysis byproducts into the culture media (Fig. 1 and Fig. S1). RGCs were stimulated at 37°C and 5% CO_2_ for 4 h continuously. Electrical stimulation was applied using an Arbitrary Waveform Generator (RIGOL DG 822 2-Channel AWG, Portland, OR, 97,223) with a Dual-Channel High-Voltage Wideband Amplifier (TEquipment, Long Branch, NJ, 9200A-DST). Two tungsten needle electrodes were placed at the edges of the roof of the electrotaxis chamber to continuously measure the voltage gradient generated across the chamber. These values were recorded in MATLAB (MathWorks, Natick, MA) at 15-min intervals for the duration of the experiment using a Keysight DSOX2014A oscilloscope. To ensure that our biphasic waveforms were charge balanced, total injected charge (area under the plot, Fig. 1F) was calculated from the first and last recordings of each experiment. The total charge of the cathodic phase was divided by that of the anodic phase to give a charge balance ratio. Experiments with a charge balance ratio < 0.9 or > 1.1 were discarded. We found the ratio of the cathodic to anodic area to be 0.95 on average at the start of the experiment (± 0.05) and 0.93 at the end of the experiment (± 0.04), suggesting a slight shift in balance towards the anodic phase over time. In addition to charge balance, voltage gradient drift during the duration of the experiment was calculated from the aforementioned waveform plots and compared over time.

Time lapse images were captured with a CCD camera and the SimplePCI 5.3 imaging system (Hamamatsu Photonics, Hamamatsu City, Japan). Only axons that demonstrated *active* growth/elongation during the time-lapse videos were quantified. Axons that had formed synapses with other cells in the culture during the overnight incubation were excluded.

### Purity assay

After overnight incubation, cells were fixed with 4% paraformaldehyde for 30 min at RT. Cells were washed with 0.3% Triton X-100, then blocked with 5% horse serum for 30 min. Following this, cells were incubated overnight at 4 °C with anti-RBPMS (RNA-Binding Protein with Multiple Splicing) antibody (1:500; Millipore, Burlington MA). Cells were then washed and incubated for one hour at RT with Alexa Fluor 488–conjugated goat anti rabbit IgG (1:200; Jackson ImmunoResearch, West Grove, PA, USA; 111-545-003). Cells were then washed and incubated with Hoechst (94403; Sigma, Burlington, MA) for 10 min at RT. Images were taken using the Keyence Live Cell Imaging system BZ-X800E (Keyence, Itasca, IL) using a 10X objective. Percent of (+)-RBPMS cells of total cells was quantified; cultures were found to consist of 85% ± 3% (+)-RBPMS RGCs (n = 3, 5030 total cells quantified).

### Viability assay

Upon termination of stimulation, the Linbro plate sealer and media were carefully removed. Cells were washed twice with PBS, then stained using the LIVE/DEAD Viability/ Cytotoxicity Assay Kit (Thermo Fisher, L3224, Waltham, MA). The staining reagent was made by adding 1 ul of 4 mM calcein AM and 4 ul of 2 mM EthD-1 to 2 ml of PBS. 150 ul of solution was added to the cells and incubated in the dark at RT for 30–45 min. Images were taken using the Keyence Live Cell Imaging system BZ-X800E using a 10X objective. Total number of live and dead cells were quantified from 10 images from each culture.

### Quantification and statistics

Direction and velocity of axon growth were quantified as previously described^7^. Briefly, the electrotaxis chamber was aligned so that the EF was parallel to the horizontal axis of the image, with the cathode to the left and the anode to the right of each image. All data were collected from time-lapse videos. Images were taken at 15-min intervals and recorded for a minimum of four hours. Images were analyzed using ImageJ (NIH) or Zen (Zeiss International). Direction and speed of axon growth were quantified by tracing the movement of its growth cone from the time-lapsed videos as previously described^7,14^.

As measured by the angle function on Zen (Zeiss International), an axon was deemed to be growing towards the cathode if its observed growth was within 120 degrees of the negative electrode, and towards the anode if within 120 degrees of the positive electrode. The remaining axons were deemed to be growing perpendicular to the EF. Because growing axons extend, retract, and “wobble” by approximately 10°, directionality was assigned as the average angle of growth that was observed during the 4 h of videography (e.g., if an axon wobbled between 140 and 150 degrees, an angle of 145 was assigned). Axon length was measured by tracing the segment of each axon demonstrating active growth during the experiment–that is, from the location of the growth cone at the beginning of the video to its final position at the end. Total length was divided by the total amount of time active growth observed.

Results are reported from at least three independent tissue culture experiments performed with pups born from at least three separate litters and from different mating pairs. The percentage of total axons growing towards the cathode, anode, and perpendicular to the EF, and average axon speed (± standard deviation (SD)) are reported unless otherwise stated. Significant differences between cultures were determined using one-way analysis of variance (ANOVA) followed by Tukey’s post hoc test for multiple comparisons using GraphPad Prism 7 (San Diego, CA). P < 0.05 was considered to indicate a statistically significant difference. Investigators were blinded to experimental conditions when quantifying the results.

## Results

### RGC axons exhibit cathode-directed growth in the presence of DC

**Figure 2:**
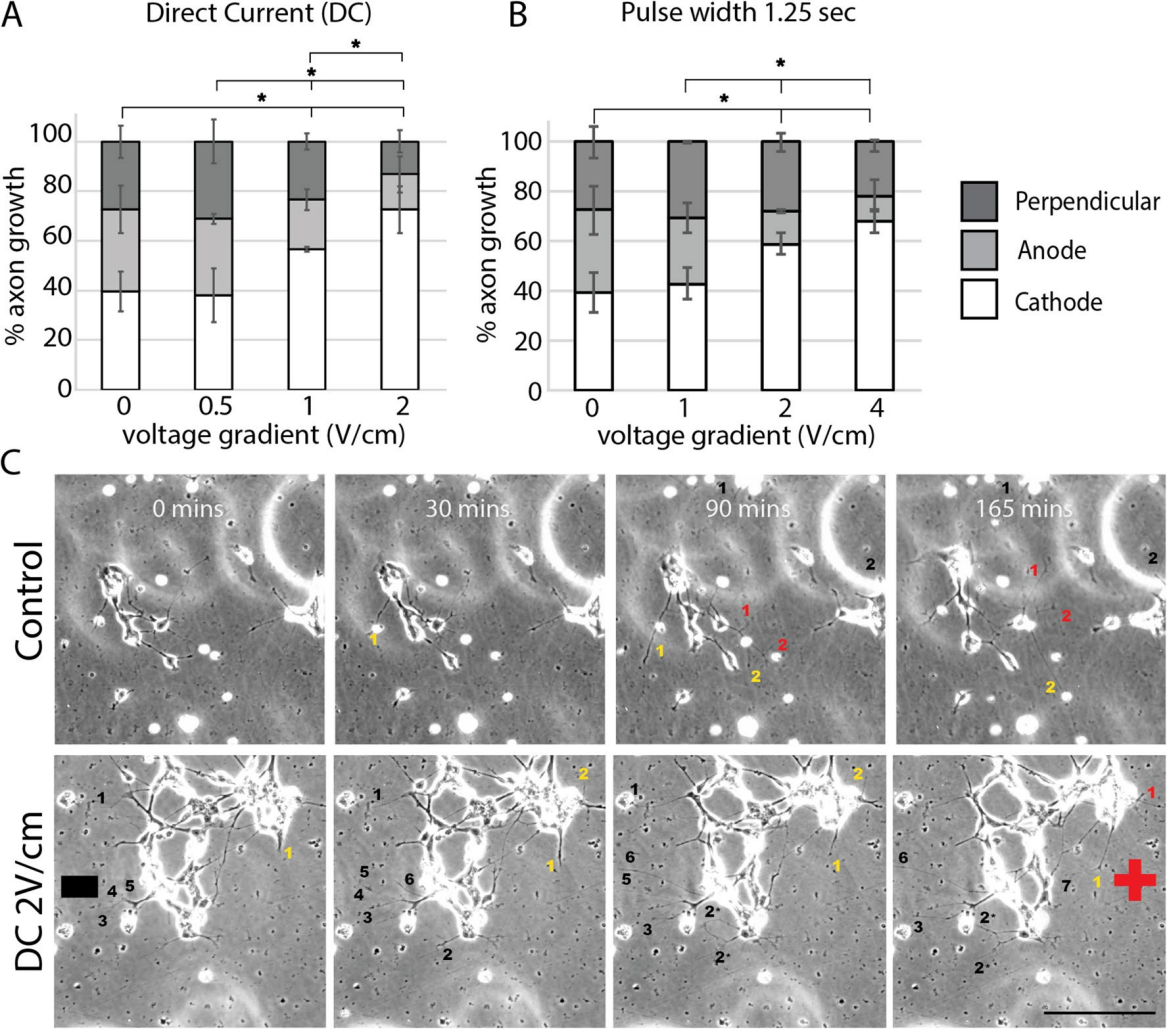
Larger pulse amplitudes direct more avid cathodic growth of RGC axons. Purified RGC cultures were exposed to direct current (**A**) or monophasic pulse stimulation (**B**). Increases in pulse amplitude led to increases in cathodic-directed growth of RGC axons (*p < 0.05 comparing cathodic growth; ANOVA followed by Tukey’s multiple comparisons). Tissue culture experiments were performed concurrently and only segregated into separate charts to facilitate flow of the paper. (**C**) Stills of time lapsed recordings of RGC axon growth in control and 2 V/cm DC treated cultures. Black numbers label axons that grew towards the cathode, red numbers label axons that grew towards the anode, while yellow numbers label axons that grew perpendicular to the EF. Scale bar 100 µm.

Previously, we demonstrated that RGC axons grow towards the negatively-driven electrode (cathode) when direct current (DC) is applied, corresponding to EFs of 1 V/cm or 2 V/cm in amplitude^7^. However, these previous experiments were performed on full thickness retinal explants which contain a heterogeneous collection of cells including RGCs, photoreceptors, and bipolar neurons. To demonstrate that DC acts directly on RGCs, we exposed purified RGCs to DC. RGCs were isolated from post-natal day 0 to 1 mouse retina as these RGCs more readily sprout new axons in culture. In cultures exposed to 1 V/cm or 2 V/cm DC, we observed significantly more cathode-directed growth compared to unstimulated controls (Fig. 2A,C, Fig. S2A), similar to our prior retinal explant experiments^7^. Notably, no significant difference was noted between control cultures and cultures exposed to 0.5 V/cm DC (Fig. 2A, Fig. S2A), indicating a minimum stimulation amplitude is needed to affect axon growth.

### RGCs exhibit cathode-directed growth in the presence of monophasic pulse stimulation

Although effective at directing axon growth, DC (Fig. 1B) cannot be applied to living organisms at high voltages or for prolonged periods of time without inducing tissue or electrode damage from charge accumulation and irreversible chemical reactions^8^. A safer alternative is alternating current (AC; Fig. 1C) which pairs a positive electrical pulse with a symmetric negative pulse. Although AC is charge balanced and thus safer for clinical application than DC, previous work by us has shown that retinal axons acutely redirect their growth towards the “new” cathode when EF polarity is switched^7^. In other words, the symmetry of the cathodic and anodic pulses in AC waveforms would essentially “cancel out” their effect on RGC axon growth and lead to net zero directional growth. Given this, we hypothesized that a hybrid waveform in which the cathodic pulse is paired with an *asymmetric* anodic pulse could be both (i) safe, if charged balanced, and (ii) effective at directing axon growth, if the right amount of asymmetry is maintained between the amplitudes and pulse widths of each pulse (ACB; Fig. 1D). To determine the optimal asymmetry needed for this hybrid, asymmetric charge balanced waveform to be effective at directing axon growth, we first performed monophasic pulse stimulation experiments to determine effective stimulation parameters (Fig. 1E).

Previously, we demonstrated that neural progenitor cells migrate towards the cathode in the presence of a monophasic pulse with a 1 s pulse width, 50% duty cycle (DTC)^15^. Given this, we exposed purified RGCs to a monophasic waveform of similar duration and amplitude (2 V/cm, 400 mHz, 50% DTC or pulse width/period). As seen in Fig. 2B and S2B, RGC axons exhibited cathode-directed growth with monophasic pulsed stimulation. However, doubling the stimulation amplitude to 4 V/cm did not result in a proportional doubling in the percentage of axons that grew towards the cathode. This suggests that above a certain threshold, increasing stimulation amplitude does not have a linear additive effect on directing axon growth.

Monophasic pulse stimulation appeared to be less effective at directing axon growth in comparison to DC of the same amplitude. While axonal electrotaxis was observed with DC of 1 V/cm (Fig. 2A, Fig. S2A), this was not observed with monophasic pulse stimulation of the same amplitude (Fig. 2B, Fig. S2B). These experiments suggest that in addition to amplitude, pulse width or total injected charge may also influence the efficacy of EFs at directing axon growth.

### Minimal pulse width is necessary to direct axon growth

**Figure 3:**
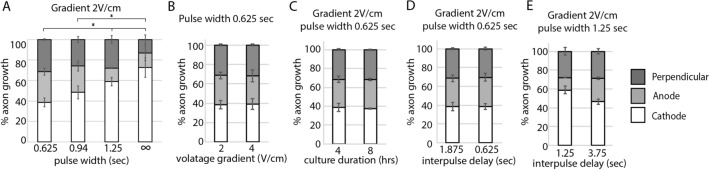
Longer pulse width duration is more effective at directing axon growth. Purified RGC cultures were exposed to monophasic EF stimulation. (**A**) Fewer RGC axons grew towards the cathode with shorter pulse widths. (**B**) Increasing voltage amplitude or (**C**) experimental duration did not increase the effectiveness of a short pulse width waveform. (**D**) Decreasing interpulse interval did not increase the effectiveness of a short pulse width waveform. (**E**) Increasing interpulse interval did not neutralize the effectiveness of a longer pulse width waveform (*p < 0.05; ANOVA followed by Tukey’s multiple comparisons).

To understand the impact of pulse width or cycle duration on directing axon growth, we exposed purified RGCs to waveforms with successively shorter pulse widths. While maintaining a stimulation amplitude at 2 V/cm, decreases in pulse width below 1.25 secs were associated with proportional decreases in electrotaxis (Fig. 3A, Fig. S3A). Interestingly, doubling the amplitude (from 2 V/cm to 4 V/cm) while maintaining a pulse width of 0.625 s duration did not compensate for the ineffective short pulse width (Fig. 3B, Fig. S3B). Even doubling the experimental time length (Fig. 3C, Fig. S3C) or decreasing the interpulse interval (Fig. 3D, Fig. S3D) so that the total charge injected into the system was equivalent to the previously effective stimulation parameters (2 V/cm, 400 mHz, 50% DTC) were insufficient to compensate for the ineffective shorter pulse width. Conversely, maintaining a pulse width at 1.25 secs, an effective pulse width length, and tripling the interpulse interval (from 1.25 to 3.75 s) did *not* significantly neutralize the electrotaxic effect of the waveform on directing RGC axon growth (Fig. 3E, Fig. S3E). These experiments demonstrate that pulse width duration, in addition to pulse amplitude, are important stimulation parameters that control cellular behavior. In fact, increasing the pulse width for ineffective amplitudes (e.g. 1 V/cm 50% DTC in Fig. 2B vs. 1 V/cm DC in Fig. 2A) rendered them effective while increasing amplitude for ineffective pulse widths (Fig. 3B, Fig. S3B) did not rescue their activity. This argues that pulse width duration exerts a *stronger* influence on EF-directed cellular behavior than pulse amplitude.

### Biphasic stimulation with ACB directs cathodic growth of RGC axons

We hypothesized that pairing monophasic pulses that are effective at directing axon growth with pulses of the opposite phase that are ineffective, would allow us to develop asymmetric charge balanced (ACB; Fig. 1D) waveforms capable of directing “net” RGC axon growth. As we are constrained by the need to develop charge balanced waveforms, the ratio of the pulse amplitude to pulse width of the cathodic pulse must be mirrored in the anodic pulse. Given that our results above demonstrated that pulse width has a stronger influence on controlling axon growth than amplitude, we prioritized longer pulse widths for our “working” pulse. With this paradigm, our “recharging” pulse has a relatively shorter pulse width but higher amplitude than our “working” pulse.

In designing our biphasic waveforms, we had the option of using cathodic-first versus anodic-first configurations. We chose to implement cathodic-first waveforms over anodic-first waveforms because prior work showed that RGC stimulation thresholds are lower with cathodic-first pulses^16^. In other words, less current would be needed to elicit a biological effect with cathodic-first waveforms.

**Figure 4:**
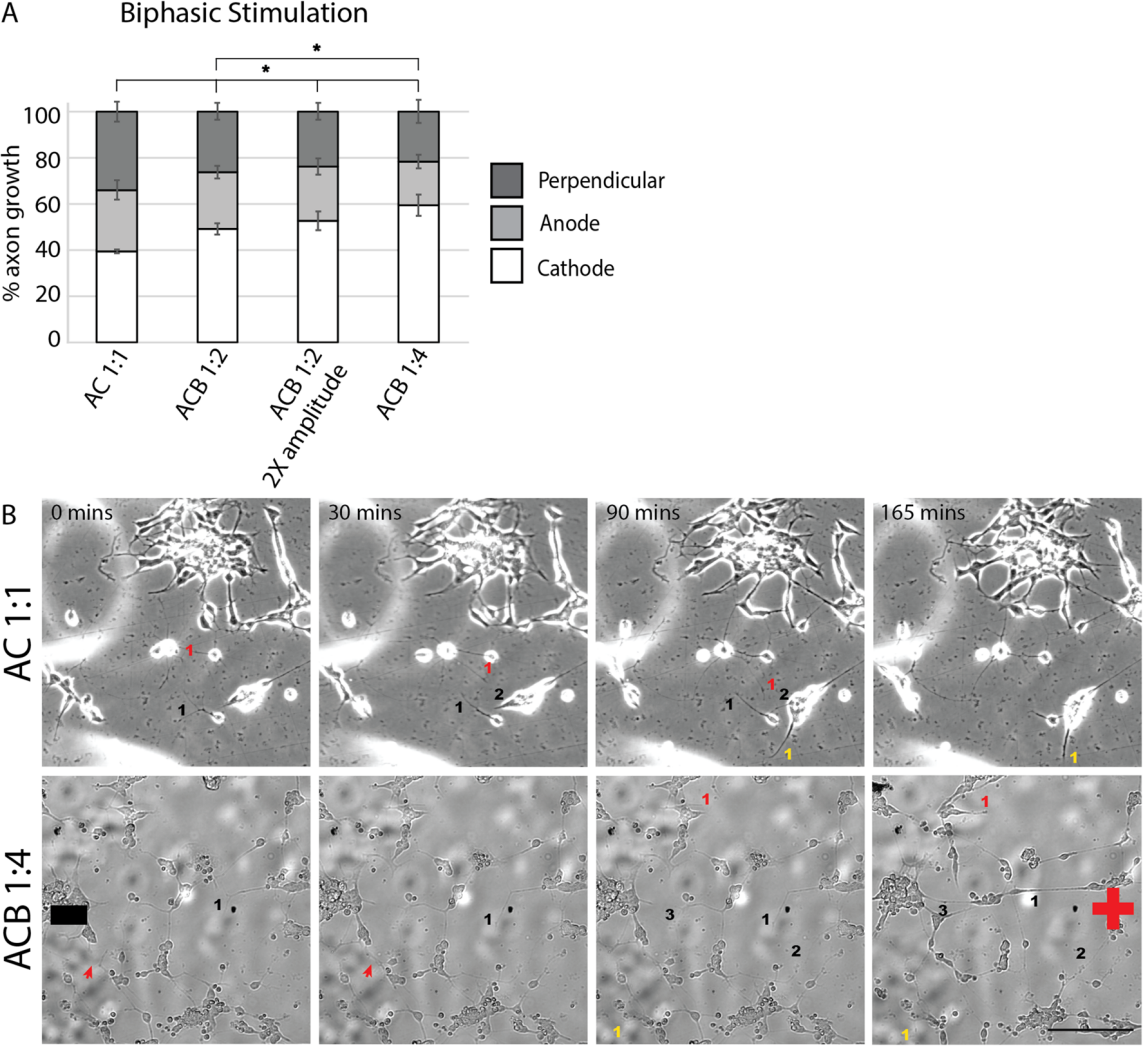
Asymmetric charge balanced (ACB) waveforms directed more avid cathodic growth of RGC axons than symmetric waveforms. Purified RGC cultures were exposed to ACB waveforms with different cathodic:anodic pulse width and pulse amplitude ratios (see Figure ***). (**A**) Increased anodic pulse width duration was associated with increases in cathodic-directed growth of RGC axons (unstimulated n = 4; AC 1:1 n = 4; ACB 1:2 n = 6; ACB 1:2 (2X-amplitude) n = 6; ACB 1:4 n = 5; * p < 0.05; ANOVA followed by Tukey’s multiple comparisons). (**B**) Stills of time lapsed recordings of RGC axon growth in AC 1:1 (phase contrast microscopy) and ACB 1:4 (DIC microscopy) treated cultures. Black numbers label axons that grew towards the cathode, red numbers label axons that grew towards the anode, while yellow numbers label axons that grew perpendicular to the EF. Scale bar 100 µm.

To test our hypothesis that ABCs are effective at directing cathodic growth of RGC axons, we paired an “effective working” (2 V/cm, 400 mHz, 50% DTC) anodic pulse with an “ineffective recharging” (− 4 V/cm, 400 mHz, 25% DTC) cathodic pulse. This 1:2 ratio ACB waveform directed significantly more cathodic growth over unstimulated controls (Fig. 4A, Fig. S4). A similar proportion of cathodic growth was also observed when we doubled the pulse amplitude of both the cathodic and anodic pulses but maintained the 1:2 cathodic:anodic ratio (a 4 V/cm, 400 mHz, 50% DTC anodic “working” pulse paired with an − 8 V/cm, 400 mHz, 25% DTC cathodic “recharging” pulse), what we termed 1:2 ratio waveform with 2X-amplitude (Fig. 4A, Fig. S4). This indicates the ratio of asymmetry rather than the absolute amplitude directs axon growth. In support of this, increasing the ratio of waveform asymmetry to 1:4 (e.g. 2 V/cm, 400 mHz, 50% DTC anodic “working” pulse paired with an − 8 V/cm, 400 mHz, 12.5% DTC cathodic “recharging” pulse) was significantly more effective at driving cathodic growth than the ACB 1:2 waveform (Fig. 4A,B, Fig. S4). Conversely, when the amplitude to pulse width ratio was maintained at 1:1 (i.e., a traditional AC current), no directional growth of RGC axons was detected over unstimulated controls (Fig. 4A,B, Fig. S4). These experiments demonstrate that the driving force behind axonal electrotaxis is the asymmetry in the duration of “working” vs. “recharging” components rather than just the presence of injected current.

### Maximum length of anodic RGC axons is shorter than cathodic RGC axons with DC stimulation

One method by which EFs could influence the direction of axon growth is by influencing the rate of neurite growth: Xenopus dorsal root ganglia neurites which faced the cathode were measured to grow faster than those that faced the anode^14^. Although there appeared to be a trend towards more rapid growth of cathode-oriented RGC axons and slowed growth of anode-oriented RGC axons treated with 2 V/cm DC stimulation, a statistically significant difference was not observed (42.6 ± 6.5 µm/h versus 25.6 ± 4.5 µm/h; p > 0.05; ANOVA followed by Tukey’s multiple comparisons; Fig. S5). The mean maximum length of anode-directed axons, however, was significantly shorter in 2 V/cm DC treated cultures compared to untreated and monophasic pulse treated cultures (Table 1; p < 0.001, ANOVA followed by Tukey’s multiple comparisons). This suggests that 2 V/cm DC stimulation negatively affects growth of anodically-oriented RGC axons without affecting growth rate.

**Table 1:** Decreased maximum length of anodically-oriented axons in DC treated cultures compared to control and monophasic treated cultures.

	Avg axon length (µm)	Avg Max axon length (µm)	Avg Min axon length (µm)
All axons			
Control	43.75 ± 7.40	146.86 ± 33.99	12.99 ± 4.43
2 V/cm DC EF	50.57 ± 7.30	148.88 ± 34.43	11.02 ± 1.08
2 V/cm 400 mHz 50% dc	50.26 ± 2.50	127.03 ± 6.77	15.04 ± 2.23
4 V/cm 400 mHz 50% dc	53.62 ± 9.25	151.85 ± 36.07	18.57 ± 7.38
Cathodic axons			
Control	47.96 ± 7.61	145.70 ± 35.59	17.26 ± 7.21
2 V/cm DC EF	55.55 ± 10.04	148.88 ± 34.42	11.84 ± 2.55
2 V/cm 400 mHz 50% dc	50.36 ± 6.74	127.03 ± 6.77	17.26 ± 2.15
4 V/cm 400 mHz 50% dc	52.87 ± 8.41	143.55 ± 47.44	19.20 ± 8.47
Anodic axons			
Control	38.76 ± 5.53	105.73 ± 15.05	14.85 ± 4.67
2 V/cm DC EF	26.89 ± 4.94	45.06 ± 29.24 ***	17.33 ± 4.67
2 V/cm 400 mHz 50% dc	46.08 ± 7.35	86.79 ± 5.76	23.16 ± 8.21
4 V/cm 400 mHz 50% dc	52.23 ± 9.31	93.95 ± 9.83	29.72 ± 6.91
Perpendicular axons			
Control	43.39 ± 11.11	94.43 ± 47.23	15.95 ± 1.39
2 V/cm DC EF	37.19 ± 6.99	81.31 ± 23.71	13.92 ± 1.13
2 V/cm 400 mHz 50% dc	49.07 ± 4.42	109.88 ± 13.48	16.31 ± 4.35
4 V/cm 400 mHz 50% dc	58.36 ± 16.00	106.79 ± 13.33	26.44 ± 8.84

Purified RGC cultures were exposed to direct current or monophasic pulse stimulation. Axon length was quantified as described in METHODS. The longest anodically-oriented axon was significantly shorter in 2 V/cm DC treated cultures over control and monophasic treated cultures. ***p < 0.001, ANOVA followed by Tukey’s multiple comparisons. Control n = 251 axons over 4 cultures; 2 V/cm DC n = 270 axons over 4 cultures; 2 V/cm monophasic n = 351 axons over 3 cultures; 4 V/cm monophasic n = 288 axons over 3 cultures.

### Preserved RGC viability with 1:4 ACB waveform stimulation

**Figure 5:**
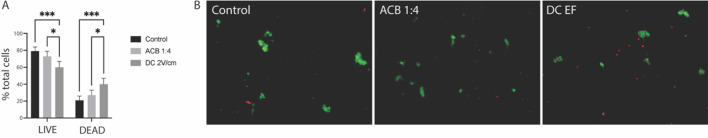
Preserved RGC viability with ACB 1:4 stimulation compared to DC EF treatment. Purified RGC cultures were exposed to ACB 1:4 or 2 V/cm DC stimulation for 4 h. (**A**) 2 V/cm DC stimulation was associated with 19% decrease in cell viability compared to controls. No significant difference was detected with ACB 1:4 stimulated cultures. (**B**) Images of LIVE/CELL viability assay. Green, living cells; red, dead cells (control n = 6533 cells over 7 cultures; 2 V/cm DC n = 5394 cells over 5 cultures; ACB 1:4 n = 2783 cells over 5 cultures; *p < 0.05, ***p < 0.001; ANOVA followed by Tukey’s multiple comparisons).

We performed LIVE/DEAD cell assays to assess the effect of EF stimulation on RGC viability. As expected, after 4 h of stimulation, DC treated cultures were associated with a significant 19% decrease in RGC cell viability compared to untreated controls (viability: 63% ± 11% DC treated cultures versus 79% ± 5% in untreated controls; p < 0.001; ANOVA followed by Tukey’s multiple comparisons; Fig. 5). Remarkably, cultures stimulated with ACB 1:4 were not associated with a significant decrease in RGC cell viability compared to untreated controls. These results demonstrate that the 1:4 ACB waveform, like DC stimulation, is effective at directing RGC axon growth but, unlike DC stimulation, does not compromise RGC viability.

### DC stimulation induces larger changes in impedance than ACB 1:4 stimulation

A possible source for the decreased viability observed with DC trials is the excessive charge accumulation produced by charge-unbalanced stimulation. Continuous injection of anodic current, such as occurs with DC stimulation, leads to production of oxidative species at the electrode surface and within the electrotaxis chamber^8^. A changing voltage profile can be used as a surrogate marker of the number of electrochemical reactions that occur from electrical stimulation and is dependent on the ionization level of the electrolyte. To compare the change in electrolyte conductivity between our different stimulation waveforms, we measured the changing voltage profile or voltage difference drift that occurred within the electrotaxis chamber over four hours of stimulation. As expected, cultures stimulated with DC demonstrated a large drift in voltage difference, with an average change of − 46% ± 13.5% (n = 5). This was significantly higher than the − 10.5% ± 6.6% (n = 5) drift seen in unstimulated cultures. Cultures stimulated with the 1:4 ACB waveform demonstrated an average of − 15.7% ± 7.1% (n = 9) drift which did not differ significantly from the drift measured in untreated control cultures. Together, our measurements demonstrate that ACB waveforms lead to less charge accumulation and byproducts from electrolysis than DC, which may account for the improved viability seen in ACB versus DC stimulation.

## Discussion

Development of electric field application into a technology to enhance endogenous repair mechanisms within the CNS to direct target-specific axon regeneration will likely require the use of biphasic waveforms that are charge balanced but also effective at directing axon growth. Towards this goal, we systematically tested monophasic waveforms with different amplitudes and pulse widths to ascertain minimal thresholds needed to design an effective asymmetric, charge balanced (ACB) biphasic waveform. Our results show that although both pulse amplitude and pulse width thresholds must be met for electrical stimulation to be effective at directing RGC axon regeneration, pulse width exerts a stronger influence on axonal growth than pulse amplitude. While monophasic waveforms with amplitudes just below threshold could be rendered effective if the pulse width was increased (e.g. monophasic stimulation with a 1 V/cm amplitude, 1.25 s pulse width, and 50% duty cycle was ineffective at directing RGC axon growth while 1 V/cm DC stimulation was effective), increasing pulse amplitude did not rescue ineffective short pulse widths (e.g. doubling the amplitude of an ineffective 0.625 s pulse width, 50% duty cycle waveform did *not* render it effective). Our findings were confirmed when the system was challenged with biphasic stimulation. Axonal electrotaxis was observed towards the cathode during the anodic phase with a longer pulse width rather than towards the cathode during the cathodic phase with the higher amplitude. No net directional growth was observed with symmetric biphasic waveform stimulation.

Importantly, our work shows that ACB waveforms are effective at directing RGC axon growth without compromising viability, a problem associated with DC stimulation. Improved viability is likely a result of ACB waveforms inducing less charge accumulation than DC.

Our finding that RGC axons grow towards the cathode during the anodic phase with ACB waveform stimulation differs from those of Babona-Pilipos et al. who demonstrated that *differentiated* neural progenitor cells (NPCs), of which RGCs resemble, did *not* exhibit electrotaxis with biphasic stimulation^17,18^. A possible explanation for our different observations is that Babona-Pilipos et al. experiments were performed to assess for cell migration of NPCs, not axon growth of mature neurons as in our experiments. The hypothesis that different waveforms may elicit different cellular responses in different cell types or that EFs of varying amplitudes can elicit different cellular responses in the same cell type has been documented previously by others^19^. Whether this finding could be exploited for clinical application, whereby different cells and different biological behaviors are targeted and activated by different stimulation frequencies, depending on need, is being actively investigated by our group and others.

The exact mechanism through which cells translate voltage gradients into directional cues and stereotypic behavior is largely unknown. Prior work suggests that extracellular EFs activate voltage-gated calcium membrane channels in a graded manner along the cell surface, translating the extracellular electrical gradient into an intracellular calcium gradient^4^. Along similar lines, our group has shown dependence of RGC axonal electrotaxis on the signaling activity of Rac Family Small GTPase 1 (Rac1), a calcium activated Rho-GTPase^7^. Activation of Rac1 in cathodically-oriented neurites, in turn, activates the actin polymerization needed for neurite elongation. Therefore, EF mediated activation of Rac1 is one possible explanation for how EFs drive and direct axon growth. Conversely, lower intra-calcium levels in anodically-oriented neurites leads to activation of Ras homolog A (RhoA), another member of the calcium dependent GTPases^14^. Unlike Rac1, RhoA induces actin depolymerization, leading to retraction of anodic neurites. It will be interesting to see if future work is able to correlate RhoA activation with a decrease in maximum neurite length of anodically-oriented axons observed in DC treated cultures (Table 1).

EFs have also been shown to redistribute charged membrane proteins including acetylcholine receptors (AchRs) and epidermal growth factor receptors on cell surfaces^4^. As AchRs are an alternative avenue for extracellular calcium can influx into a cell, this clustering of receptors may serve as an additional mechanism through which EFs can induce intracellular gradients of calcium. Regardless of the ion channel, intracellular calcium gradients likely play a major role in translating electrical gradients into directed axon growth.

Although exploiting natural cellular responses to EFs is an attractive approach to engineering tissue regenerative treatments, clinical translation is dependent on the development of safe stimulation strategies. Ultimately, biphasic stimulation will play a central role in electrical stimulation strategies aimed at promoting endogenous neurorepair.

### Supplementary Information

Supplementary Figures.

## Data Availability

Raw data can be downloaded from the following links: https://drive.google.com/drive/folders/15MlKDETd2LVUcCih5iwk0XcPmWvEhiHF?usp=share_link, https://osf.io/pa7fv/?view_only=206bd04cb3fb42da9b94112123cf9ea7. Tissue culture files are in a czi format which can be opened with Zen Black which is a freely available software produced by Carl Zeiss. The stimulation parameters and our counts for each culture are catalogued in the attached Excel sheets. The waveform measurements are txt format and can be opened and quantified using Matlab.

## References

[1] Sharf T, Kalakuntala T, Gokoffski KK (2022). Electrical devices for visual restoration. Surv. Ophthalmol..

[2] Ingvar S (1920). Reaction of cells to the galvanic current in tissue cultures. Exp. Biol. Med..

[3] Yamashita M (2013). Electric axon guidance in embryonic retina: Galvanotropism revisited. Biochem. Biophys. Res. Commun..

[4] McCaig CD, Rajnicek AM, Song B, Zhao M (2005). Controlling cell behavior electrically: Current views and future potential. Physiol. Rev..

[5] Morimoto T (2005). Transcorneal electrical stimulation rescues axotomized retinal ganglion cells by activating endogenous retinal IGF-1 system. Invest. Ophthalmol. Vis. Sci..

[6] Goldberg JL (2002). Retinal ganglion cells do not extend axons by default: Promotion by neurotrophic signaling and electrical activity. Neuron.

[7] Gokoffski KK, Jia X, Shvarts D, Xia G, Zhao M (2019). Physiologic electrical fields direct retinal ganglion cell axon growth in vitro. Invest. Ophthalmol. Vis. Sci..

[8] Merrill DR, Bikson M, Jefferys JG (2005). Electrical stimulation of excitable tissue: Design of efficacious and safe protocols. J. Neurosci. Methods.

[9] Gall C (2005). Alternating current stimulation for vision restoration after optic nerve damage: A randomized clinical trial. PLoS ONE.

[10] Kloth LC (2005). Electrical stimulation for wound healing: A review of evidence from in vitro studies, animal experiments, and clinical trials. Int. J. Low Extrem. Wounds.

[11] Percie du Sert N (2020). The ARRIVE guidelines 2.0: Updated guidelines for reporting animal research. BMJ Open Sci..

[12] Huang X, Wu DY, Chen G, Manji H, Chen DF (2003). Support of retinal ganglion cell survival and axon regeneration by lithium through a Bcl-2-dependent mechanism. Invest. Ophthalmol. Vis. Sci..

[13] Gao F (2016). Comparative analysis of three purification protocols for retinal ganglion cells from rat. Mol. Vis..

[14] Rajnicek AM, Foubister LE, McCaig CD (2006). Temporally and spatially coordinated roles for Rho, Rac, Cdc42 and their effectors in growth cone guidance by a physiological electric field. J. Cell Sci..

[15] Feng JF (2017). Electrical guidance of human stem cells in the rat brain. Stem Cell Rep..

[16] Hadjinicolaou AE (2015). Optimizing the electrical stimulation of retinal ganglion cells. IEEE Trans. Neural Syst. Rehabil. Eng..

[17] Babona-Pilipos R, Droujinine IA, Popovic MR, Morshead CM (2011). Adult subependymal neural precursors, but not differentiated cells, undergo rapid cathodal migration in the presence of direct current electric fields. PLoS ONE.

[18] Babona-Pilipos R, Pritchard-Oh A, Popovic MR, Morshead CM (2015). Biphasic monopolar electrical stimulation induces rapid and directed galvanotaxis in adult subependymal neural precursors. Stem Cell Res. Ther..

[19] Wang E, Zhao M, Forrester JV, MCCaig CD (2000). Re-orientation and faster, directed migration of lens epithelial cells in a physiological electric field. Exp. Eye Res..

