# Supplementary material for: Asymmetric charge balanced waveforms direct retinal ganglion cell axon growth
Source: Sci Rep. 2023 Aug 14;13:13233. doi: 10.1038/s41598-023-40097-6 (PMC10425404; doi:10.1038/s41598-023-40097-6)
Supplement: Supplementary file 1 — Supplementary Figures. [file 41598_2023_40097_MOESM1_ESM.docx]

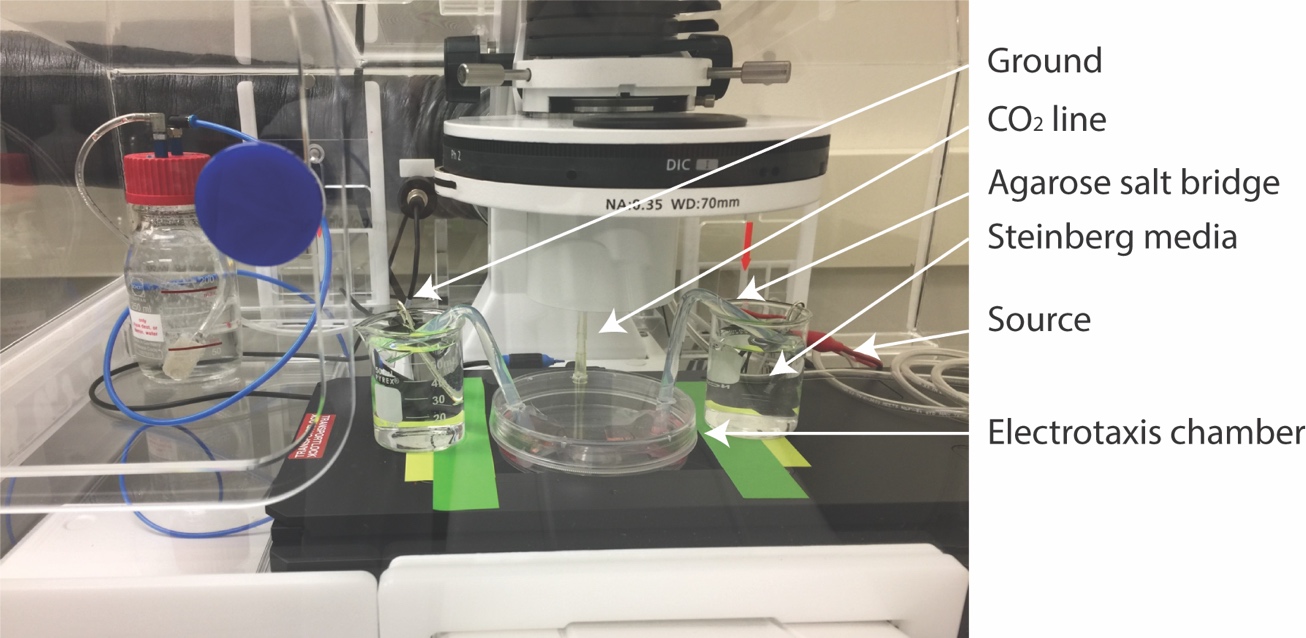


**Figure S1. Electrotaxis Chamber**

An electrotaxis chamber was built onto a 100 mm tissue culture plate which was then placed inside a gas/temperature chamber-controlled inverted Axio Observer 7 microscope. Agarose salt bridges were used to deliver current to the electrotaxis chamber.


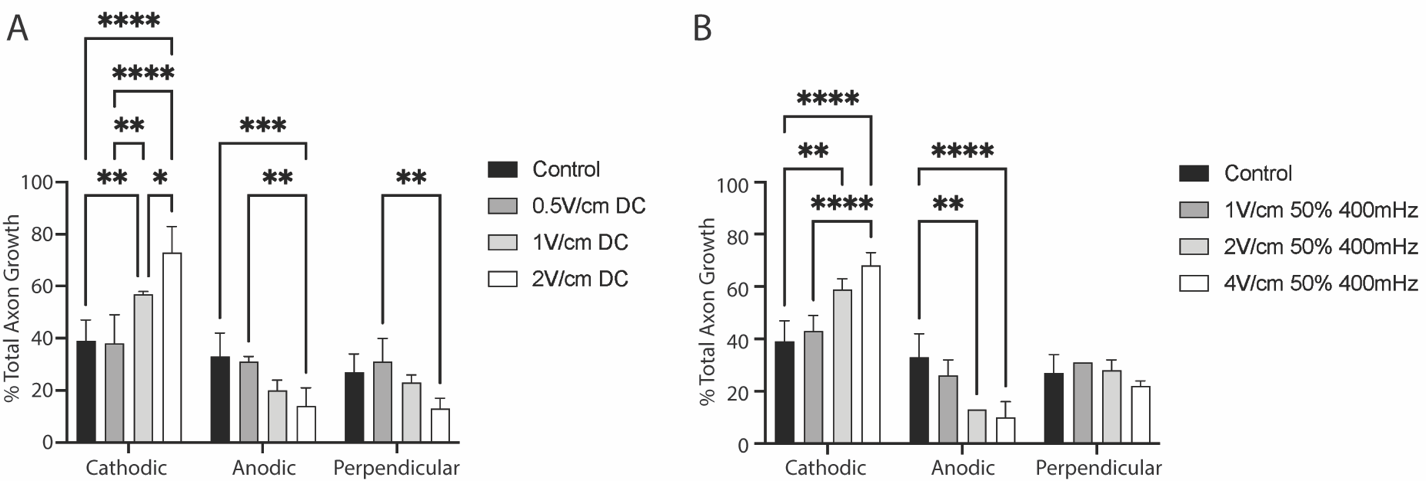


**Figure S2. Larger pulse amplitudes direct more avid cathodic growth of RGC axons.** Purified RGC cultures were exposed to direct current (A) or monophasic EF stimulation (B). Increases in pulse amplitude led to increases in cathodic-directed growth of RGC axons. * p < 0.05; ** p < 0.01; *** p < 0.001; **** p < 0.0001; ANOVA followed by Tukey’s multiple comparisons. Control n = 251 axons over 4 cultures; 0.5 V/cm DC n = 559 axons over 3 cultures; 1 V/cm DC n = 368 axons over 3 cultures; 2 V/cm DC n = 270 axons over 4 cultures; 1 V/cm monophasic n = 510 axons over 3 cultures; 2 V/cm monophasic n= 351 axons over 3 cultures; 4 V/cm monophasic n = 288 axons over 3 cultures. Tissue culture experiments were performed concurrently and only segregated into separate charts to facilitate flow of the paper.


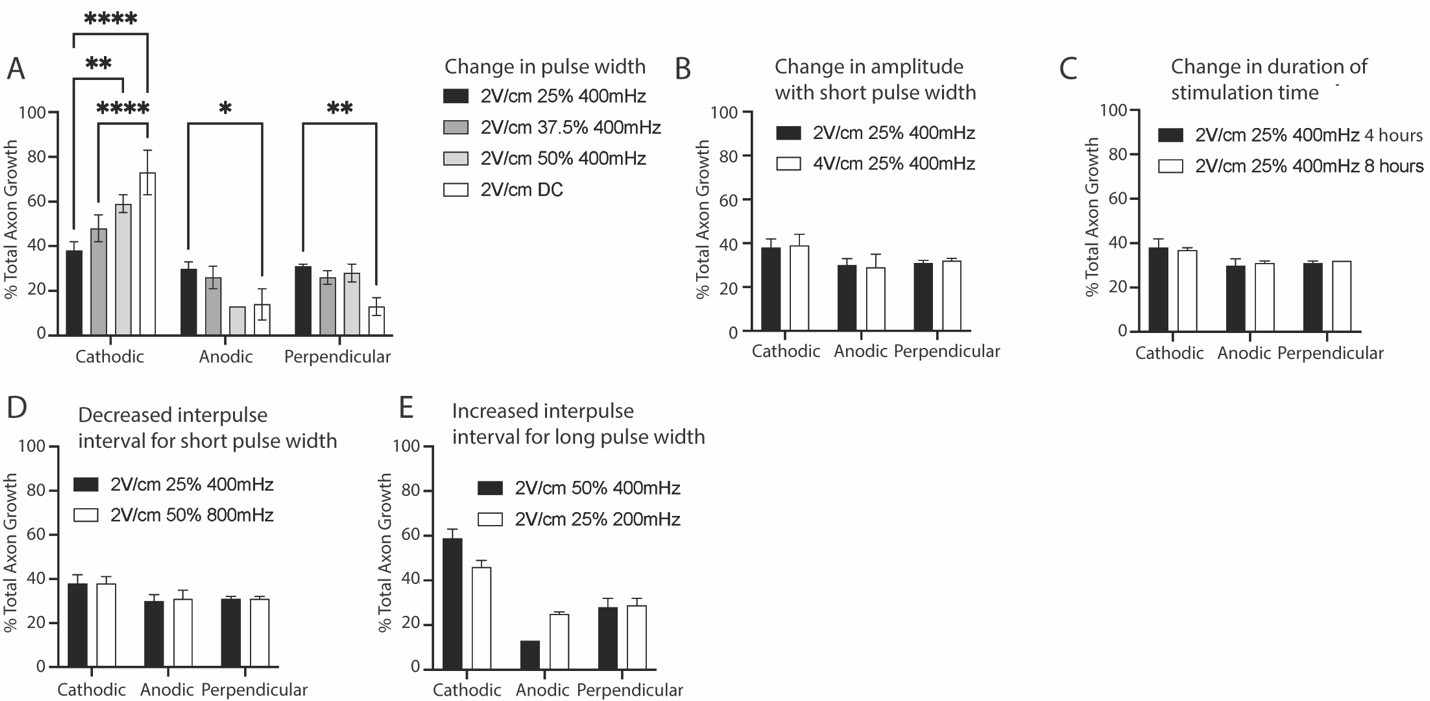


**Figure S3. Longer pulse width duration is more effective at directing axon growth.** Purified RGC cultures were exposed to monophasic EF stimulation. (A) Fewer RGC axons grew towards the cathode with shorter pulse widths. (B) Increasing voltage amplitude or (C) experimental duration did not increase the effectiveness of a short pulse width waveform. (D) Decreasing interpulse interval did not increase the effectiveness of a short pulse width waveform. (E) Increasing interpulse interval did not neutralize the effectiveness of a longer pulse width waveform (n=4; * p < 0.05; ** p < 0.01; *** p < 0.001; **** p < 0.0001; % denotes duty cycle; ANOVA followed by Tukey’s multiple comparisons).

(A) 2 V/cm 25% duty cycle n = 396 axons over 3 cultures; 37.5% duty cycle n = 316 axons over 3 cultures; 50% duty cycle n = 351 axons over 3 cultures; 100% duty cycle n = 270 axons over 4 cultures.

(B) 2 V/cm n = 458 axons over 3 cultures; 4 V/cm n = 288 axons over 3 cultures.

(C) 4 hours n = 458 axons over 3 cultures; 8 hours n = 508 over 3 cultures.

(D) 400 mHz n = 396 axons over 3 cultures; 800 mHz n = 444 axons over 4 cultures

(E) 400 mHz n = 351 axons over 3 cultures; 200 mHz n = 611 axons over 4 cultures


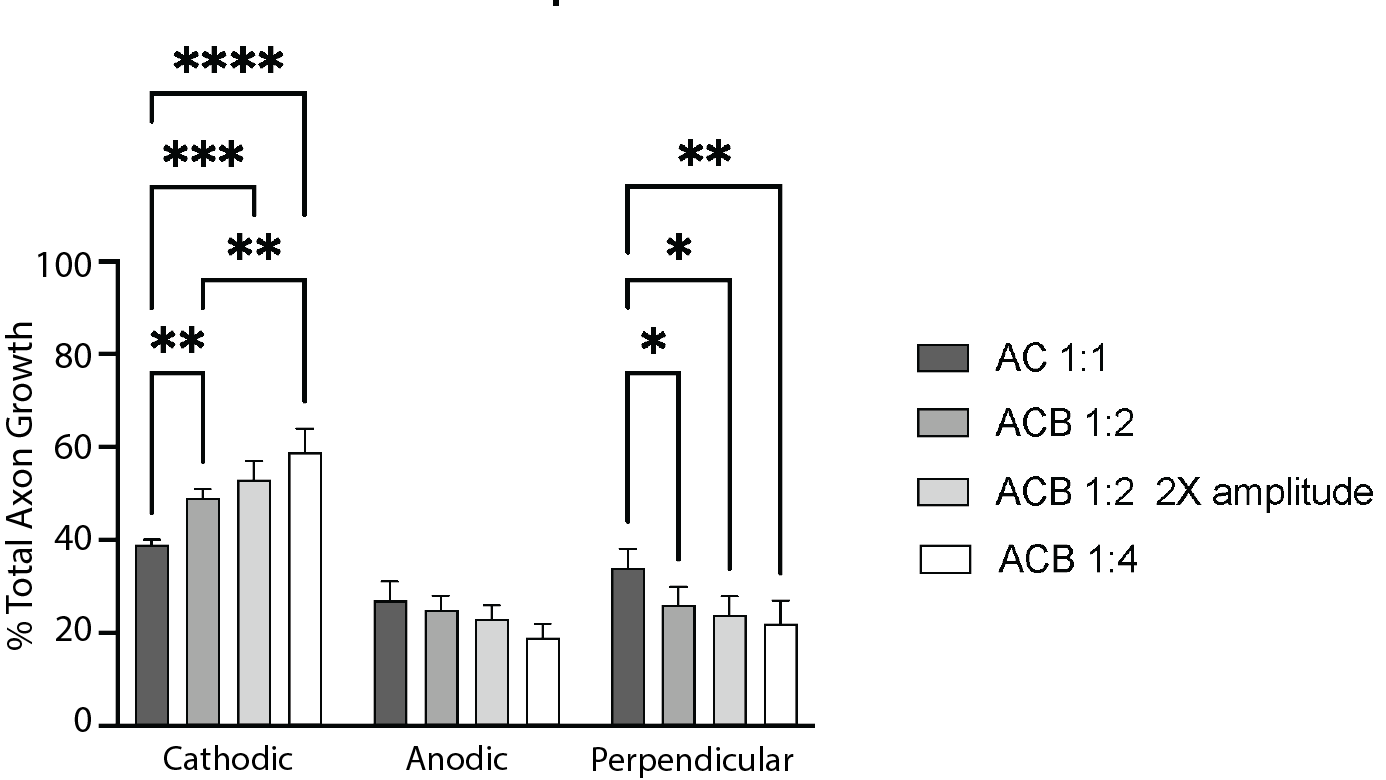


**Figure S4. Asymmetric charge balanced (ACB) waveforms directed more avid cathodic growth of RGC axons than symmetric waveforms.** Purified RGC cultures were exposed to ACB waveforms with different cathodic:anodic pulse width and pulse amplitude ratios. Increasing anodic pulse width duration was associated with increases in cathodic-directed growth of RGC axons (control n = 251 axons over 4 cultures; AC 1:1 n = 403 axons over 4 cultures; ACB 1:2 n = 696 over 6 cultures; ACB 1:2 (2X-fold amplitude) n = 579 axons over 6 cultures; ACB 1:4 n = 467 axons over 5 cultures; * p < 0.05; ** p < 0.01; *** p < 0.001; **** p < 0.0001; ANOVA followed by Tukey’s multiple comparisons).


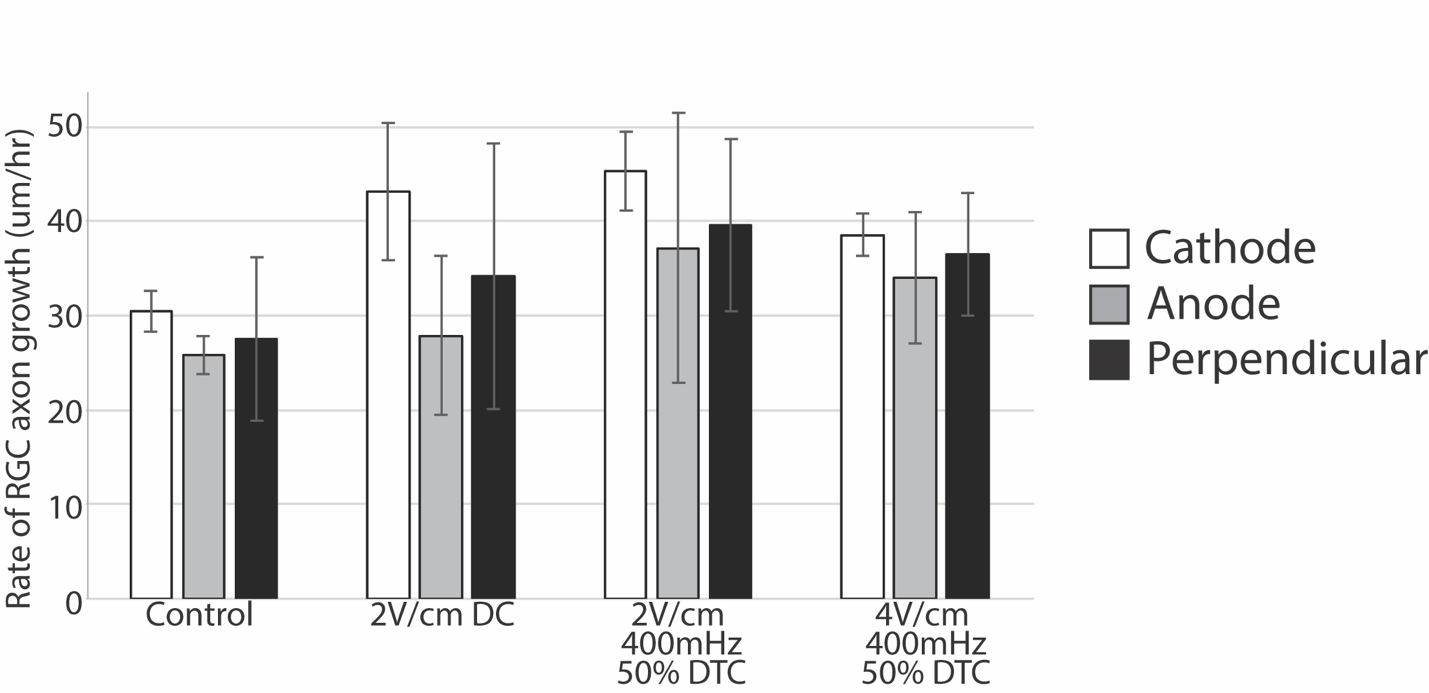


**Figure S5. No difference in rate of RGC axon growth with EF treatment.** Purified RGC cultures were exposed to direct current or monophasic EF stimulation. Rate of axon growth was quantified as described in METHODS. No significant difference was detected in cathodic- or anodic-oriented RGC axons in DC EF or monophasic treated cultures, ANOVA followed by Tukey’s multiple comparisons. dc, duty cycle. Control n = 251 axons over 4 cultures; 2 V/cm DC n = 270 axons over 4 cultures; 2 V/cm monophasic n= 351 axons over 3 cultures; 4 V/cm monophasic n = 288 axons over 3 cultures. Table lists average RGC axon length, average maximum RGC axon length, and average minimal RGC axon length in different culture conditions. DTC, duty cycle.
